# Prediction of formation abrasiveness under the action of a PDC bit based on the fractal dimension of a rock surface

**DOI:** 10.1038/s41598-024-67521-9

**Published:** 2024-07-16

**Authors:** Changhao Wang, Shaojie Li, You Li, Lidong Hou, Jinsong Bai, Qianwen Feng, Zhaoyi Liu

**Affiliations:** 1 National Key Laboratory of Multi-Resource Collaborative Green Exploitation of Continental Shale Oil, Northeast Petroleum University, Daqing, 163318 China; 2 Liaohe Oilfield Oil Production Technology Research Institute, Panjin, 124010 China; 3 Heli (Tianjin) Energy Technology Co., Ltd., Tianjin, 300452 China

**Keywords:** Rock abrasiveness, Drill bit wear, PDC compact, Surface fractal dimension, Energy science and technology, Engineering

## Abstract

During rock drilling, a drill bit will wear as it breaks the rock. However, there is no uniform grading standard for rock abrasiveness. To solve this problem, the wear mechanisms of a polycrystalline diamond compact (PDC) bit and the formation it is drilling into are analyzed in depth, and an abrasiveness evaluation method based on the fractal dimension of the rock surface topography is established. Initially, a three-dimensional digital model is generated from a scanning electron microscope image of the rock after drilling; next, an evaluation of the irregularities on the rock surface is performed using an adapted Weierstrass–Mandelbrot (W-M) function to ascertain the fractal dimensionality. Then, the microcontact characteristics of the contact surface between the formation and the PDC bit are analyzed, and the distribution of the microconvex contact points of the two-body friction pair in a region is obtained. Because the sliding friction between the drill bit and the rock produces a large amount of heat, according to the contact area formula of the friction surface and heat conduction theory, the temperature rise and overall temperature distribution of the formation and PDC bit under the condition of sliding friction are revealed, and the real contact area between the formation and the drill bit within a certain temperature range is obtained. Finally, the evaluation index of rock abrasiveness under sliding conditions is established by adopting the wear weight loss of the rock cutting tool per unit volume as the index of rock abrasiveness, and the model is verified by a microdrilling experiment. The research in this paper is highly important for improving the rock-breaking efficiency and bit service life during drilling.

## Introduction

At present, polycrystalline diamond compact (PDC)bits are the most widely used rock breaking tool in oil and gas exploration and development, and their drilling footage accounts for more than 85% of all drilling footage in this industry^1^. However, with the continuous advancement of exploration, an increasing amount of new reserves are being produced from deep wells, ultradeep wells and other areas where mining is more difficult^2^. During the drilling process in these strongly abrasive formations, the PDC bit will wear when it breaks the rock. As the PDC cutter scrapes and breaks the rock, it rubs violently against the cuttings and rock. The local high temperature generated by this process accelerates the wear failure of the PDC cutter, which not only greatly shortens the service life of the whole drill bit but also reduces the rock-breaking efficiency of the drill bit. The ability of rock to wear a drill bit is called rock abrasiveness. However, due to the complexity of the PDC bit wear process, there is a lack of widely accepted general mathematical models for accurately describing PDC tool wear; therefore, there is no uniform evaluation index for rock abrasiveness.

Various scholars have utilized a range of experimental methodologies and measurement indicators to forecast the abrasiveness of rocks. The experimental materials employed include core samples, rock blocks, rock powder, and rock cuttings, with grinding tools such as metal needles, metal drill bits, metal rings, and microdrill bits^3^. The Cerchar abrasivity index (CAI) test, developed by the International Society for Rock Mechanics and Rock Engineering (ISRM), is a widely utilized experimental method for assessing rock abrasiveness^4^. This test involves placing a steel needle with a Vickers hardness of 610 and a tensile strength of 200 kg/mm^2^ in contact with the rock surface under a load of 68.646 N and subsequently sliding it for 1 s to generate a 10 mm long groove. The abrasiveness index of the rock is determined by quantifying the wear experienced by the steel needle. Moradi^5^ suggested a new method to estimate disc cutter wear using scaled-down rock cutting tests, improving the accuracy of rock abrasivity classification. This method distinguishes normal wear from disc deformation and introduces new abrasivity indices for easier and more precise comparisons of test results and rock classification. Wang^6^ tested granite samples at various temperatures and cooling rates using the CAI test. The results indicated that the CAI value increased in the first few millimeters of the scratch distance and was affected by indentation in subsequent distances. Another method for measuring abrasivity was proposed based on P-wave velocity and acoustic emission, and a 10 mm scratch distance was recommended for accurate measurements of highly fragmented rocks. Li^7^ created a prediction model using a back propagation–artificial neural network (BP-ANN)by processing rock sample data via normalization, correlation analysis, and grouping. The model is used to describe how the mineral content and particle size affect rock abrasiveness and hardness. Zhang^8^ elucidated the grinding energy per unit volume of rock using a worn flat-bottom diamond bit, employing the principles of mechanical balance and energy conservation in the rock drilling process. Two optimal regression models were developed through stepwise multiple regression analysis to predict the CAI, incorporating the uniaxial compressive strength and grinding energy as variables. The models achieved a prediction accuracy of 96%.

The analysis of tribological wear involves investigations at both the microscale and macroscale. Previous methodologies predominantly utilized empirical statistical and numerical techniques, neglecting the influence of rock surface topography on drill bit deterioration. This study examines the sliding friction characteristics of PDC drill bits during interactions with rock by utilizing fundamental tribological principles. A microcontact model is introduced for the drill bit–rock friction interface, integrating the fractal dimensions of the rock surface. This model can be used to perform a comprehensive analysis of the wear mechanisms affecting PDC drill bits and thus is a new methodological framework for evaluating the wear on PDC bit cutters.

## Fractal features of rock surface morphology

### Composition of rough surfaces

**Figure 1:**
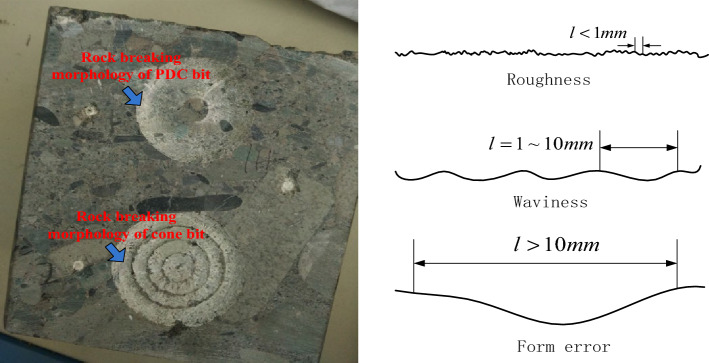
The surface morphology of rock broken by different drill bits.

Figure 1 displays an example of the complex surface structure of a rock caused by a drill bit, with irregular geometric shapes made up of peaks and valleys of varying heights and spacings^9^.

Surface morphology can be categorized into roughness, waviness, and form error based on wave peak and wavelength differences. The traditional definition considers a form error with a peak-to-valley distance less than 1 mm as the surface roughness^10^. This work focuses on surface roughness, waviness, and form error, with waviness referring to periodic peaks and valleys with wave distances between 1 and 10 mm and form error referring to fluctuations in peak-valley wave distances greater than 10 mm.

To understand wear modes, studying wear formation, change, and failure mechanisms at the microscale is essential. This paper focuses on the wear mechanisms of PDC bits at the microscale, where the surface waviness and form error of the rock have minimal impacts on the wear characteristics. Characterization methods for rock surface roughness are discussed in the following section.

### Characterization method of rock surface fractal features

Different parameters are used to describe the surface roughness of rock, including one-dimensional, two-dimensional, and three-dimensional characteristics. These parameters, such as the arithmetic mean deviation of the contour and skewness of the height distribution, are commonly used to analyze contact mechanics and wear surfaces in friction pairs.

In the past, surface roughness characterization was limited by the measurement length range L, which did not fully capture changes in surface morphology across the entire contact surface. The sampling interval size significantly impacts the resulting histogram and distribution curve when analyzing surface morphology features. Spatial evaluation parameters such as the covariance function $V\left( \tau \right)$ or autocorrelation function $R\left( \tau \right)$ can show the relationship between adjacent contour peaks and the curve's change trend. The autocovariance is the average product of the contour heights $z\left( x \right)$ and $z\left( {x + \Delta \tau } \right)$ at a distance $\Delta \tau$.

1 $$ V\left( {\vartriangle \tau } \right) = \mathop {\lim }\limits_{L \to \infty } \frac{1}{L}\int_{0}^{L} {z\left( x \right)} z\left( {x + \vartriangle \tau } \right){\text{ d}}x $$

The autocovariance function is normalized, and the covariance is calculated by the difference between the contour curve and the midline so that the autocorrelation function $R\left( {\Delta \tau } \right)$ is obtained:

2 $$ R\left( {\vartriangle \tau } \right) = \mathop {\lim }\limits_{L \to \infty } \frac{1}{{L\sigma^{2} }}\int_{0}^{L} {\left[ {z\left( x \right) - m} \right]\left[ {z\left( {x + \vartriangle \tau } \right) - m} \right]{\text{ d}}x} = \frac{{V\left( {\vartriangle \tau } \right) - m^{2} }}{{\sigma^{2} }} $$

The autocorrelation function is crucial for analyzing changes in the surface morphology of rocks and drill bits by distinguishing between periodicity and randomness to understand contour shape characteristics^11^. Fractal surfaces are characterized by their fractal dimension and scale coefficient, which are calculated using different computational methods. The fractal dimension is determined by a specific power law formula:

3 $$ M\left( \tau \right) = G\tau^{D} $$

Within this formula, $M\left( \tau \right)$ represents the contour curve's measure, $\tau$ denotes the scaling measure used, $D$ signifies the fractal dimension reflecting the contour curve's similarity and complexity, and $G$ is the scale coefficient, indicating the contour's amplitude.

### Three-dimensional numerical reconstruction of the rock surface morphology

**Figure 2:**
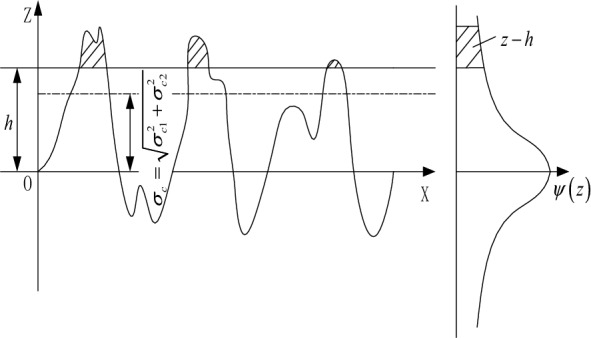
Contact surface between the drill bit and the formation.

To determine the fractal dimension and scale coefficient indicative of rock surface roughness, an analysis of the microscale morphological features of rock was performed. Figure 2 displays a scanning electron microscopy (SEM) image of a rock surface magnified 3000 times. The pixel's grayscale, heavily influenced by the actual morphological features, accurately mirrors the rock's surface roughness, establishing a direct link with its morphology, and the gray value data can be contoured. The image was reconstructed in three dimensions using MATLAB by utilizing gray value data.

The gray image $f\left( {x,y} \right)$ of a rock surface exists in the form of a matrix and can be used for computations. Its mathematical form is:

4 $$ f\left( {x,y} \right) = \left[ {\begin{array}{llll} {f\left( {0,0} \right)} & {f\left( {0,1} \right)} & \cdots & {f\left( {0,m - 1} \right)} \\ {f\left( {1,0} \right)} & {f\left( {1,1} \right)} & \cdots & {f\left( {1,m - 1} \right)} \\ \vdots & \vdots & \vdots & \vdots \\ {f\left( {n - 1,0} \right)} & {f\left( {n - 1,1} \right)} & \cdots & {f\left( {n - 1,m - 1} \right)} \\ \end{array} } \right] $$

Leveraging fractal geometry theory, a mathematical model was developed to derive the fractal dimension and scale coefficient that represent the surface morphology of rock from the gray value data of SEM images^12^.

For each pixel in the SEM image's grayscale representation of the specimen surface, the physical position coordinates and the grayscale intensity, denoted as $Z_{i}$, together form a stochastic dataset:

5 $$ \left[ {\left( {x_{i} ,y_{i} } \right);z_{i} } \right],\;\;\;\;\;i = 1,2,3, \ldots ,N $$

Based on the dataset, the autocorrelation function for the grayscale values is computed using formula (2). A natural logarithm is applied to $S\left( {\tau_{i} } \right)$ and $\tau_{i}$ for linear regression, leading to the calculation of the slope a and the y-intercept b of the resulting line.

The scale coefficient of rock surface roughness is^13^:

6 $$ G = \exp \left\{ {\frac{{b + \ln \left\{ {2\Gamma \left( {5 - 2D} \right)\sin \left[ {\left( {2 - D} \right)\pi } \right]} \right\} - \ln \pi }}{{2\left( {D - 1} \right)}}} \right\} $$

Currently, for surfaces exhibiting multiscale and self-affine properties, the Weierstrass–Mandelbrot (W-M) fractal function is extensively utilized to describe surface roughness across scales ranging from nanometers to millimeters. This function is known for its continuity, nondifferentiability, and self-affinity, making it an ideal representation of random surface contours. Its function can be constructed as:

7 $$ g\left( x \right) = \sum\limits_{n=- \infty }^{\infty } {\lambda^{{\left( {D - 2} \right)n}} } \left[ {1 - \cos \left( {\lambda^{n} x} \right)} \right] $$

where $\lambda$ is a constant greater than 1, $D$ is the fractal dimension, $1 < D < 2$, and $n$ is the number of natural sequences.

Bhushan et al.^14^ obtained the modified W-M function form as follows:

8 $$ z\left( x \right) = G^{D - 1} \sum\limits_{{\omega = \omega_{L} }}^{{\omega_{U} }} {\frac{{\cos \left( {2\pi \omega x} \right)}}{{\omega^{2 - D} }}} $$

where $z\left( x \right)$ is the surface contour; $x$ is the contour measurement coordinate; $\omega_{L}$ and $\omega_{U}$ are the low-frequency and high-frequency upper limits of the contour, respectively; and $\omega = b^{\prime n}$, where $b^{\prime}$ is a constant greater than 1 and is generally taken as $b^{\prime} = 1.5$.

If the mean square error of a simulated contour curve is $\sigma$, then the scale coefficient can be determined by the following formula:

9 $$ G^{D-1} = 2\sigma \left( {\omega_{L} \omega_{U} } \right)^{2-D} \sqrt {\frac{2 - D}{{\omega_{U}^{4 - 2D} - \omega_{L}^{4 - 2D} }}} $$

In the actual calculation process, the arithmetic mean square error of the rock surface contour is easier to obtain. For a given real rock surface morphology, after determining its self-affine fractal dimension $D$, $G$ can be obtained by substituting the mean square roughness $\sigma$ measured by the instrument into formula (9). At this time, the rough surface can be described more accurately based on formula (8).

## Friction analysis of the formation–PDC bit contact surface under thermal–mechanical coupling

### The real contact area between the formation and the PDC bit

The hypothesis here is that the characteristics of surface asperities can be encapsulated by formula (8), with the surface roughness exhibiting statistical isotropy. This leads to a simplification where the interaction between the geological formation and the rough surface of the drill bit is modeled as the interaction between a rough surface and a perfectly smooth surface. The root mean square roughness values of the two surfaces are $\sigma_{c1}$ and $\sigma_{c2}$, respectively, where $h$ represents the separation of their centerlines. Thus, the interaction between the hard particles of the rock and the drill bit surface is reinterpreted as an encounter between a smooth elastic surface and another rough rigid surface with a root mean square roughness of $\overline{\sigma }_{c} = \sqrt {\sigma_{c1}^{2} + \sigma_{c2}^{2} }$, as depicted in Fig. 2.

It is assumed that the contact points have different sizes and are randomly distributed on the contact surface. If the area distribution of the contact points is $n\left( a \right)$, then $n\left( a \right){\text{d}}a$ is equal to the number of contact points with an area between $a$ and $a + {\text{d}}a$. The real contact area is:

10 $$ A_{r} = \int_{{a_{s} }}^{{a_{l} }} {n\left( a \right)} {\text{d}}a $$

where $a_{s}$ and $a_{l}$ are the minimum and maximum contact areas, respectively.

When the deformation $\delta$ at the top of the asperity is greater than the critical deformation $\delta_{c}$, the deformation transforms from elastic to plastic behavior:

11 $$ \delta_{c} = \left( {\frac{{\pi K\sigma_{y} }}{{2E^{ * } }}} \right)^{2} R $$

where $\sigma_{y}$ is the yield strength of the PDC cutter; $E^{ * }$ is the comprehensive elastic modulus of the two contact surfaces; $K$ is the correlation coefficient between the cutting tooth hardness $H$ and yield strength $\sigma_{y}$, $K = \frac{H}{{\sigma_{y} }}$; and $R$ is the radius of curvature at the top of the asperity, which depends on the area $a$ of the contact point. The latter corresponds to the contact length $l$ on the contour line, that is:

12 $$ l = a^{{{1 \mathord{\left/ {\vphantom {1 2}} \right. \kern-0pt} 2}}} $$

**Figure 3:**
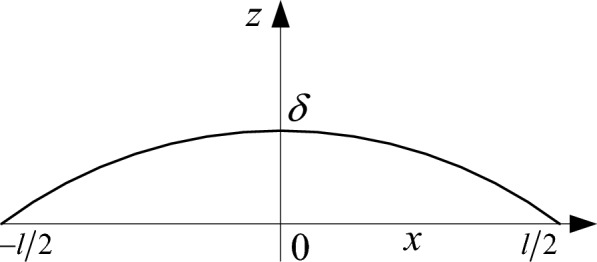
The geometry of a contact point of length $l$.

The geometric shape of the contact point with length $l$ is shown in Fig. 3, and the critical contact area $a_{c}$ is derived as follows:

13 $$ a_{c} = \frac{{G^{2} }}{{\left( {{{K\sigma_{y} } \mathord{\left/ {\vphantom {{K\sigma_{y} } {2E^{ * } }}} \right. \kern-0pt} {2E^{ * } }}} \right)^{{{2 \mathord{\left/ {\vphantom {2 {\left( {D - 1} \right)}}} \right. \kern-0pt} {\left( {D - 1} \right)}}}} }} $$

If $a < a_{c}$, plastic deformation occurs; if $a > a_{c}$, elastic deformation occurs.

When the peak contact area $a_{l}$ on the formation–bit interface is within the bounds of the critical elastic microcontact area $a_{ec}$ and critical plastic deformation microcontact area $a_{pc}$, certain contact asperities undergo elastic‒plastic deformation, while others experience purely plastic deformation. According to the principles of elastic‒plastic mechanics^15^, the total load $F$ on an asperity equals the sum of the elastic load $F_{ep}$ and the plastic load $F_{pp}$. By integrating, one can correlate the actual contact area of the formation–bit friction pair with the axial load on the bit, as detailed in formula (14).

14 $$ \begin{aligned} & p^{ * } = 1.1K_{f} \phi_{c} A_{r}^{ * } - K\left( D \right)g_{1} \left( D \right)A_{r}^{{ * \frac{D}{2}}} \cdot \hfill \\ & \left\{ \begin{aligned} & \frac{3}{{\left( {a_{ec}^{ * } - a_{pc}^{ * } } \right)^{2} }}\left[ {\frac{2}{6 - D}\left( {A_{l}^{{ * \left( {6 - D} \right)/2}} - a_{pc}^{{ * {{\left( {6 - D} \right)} / 2}}} } \right) - \frac{4}{4 - D}\left( {A_{l}^{{ * \left( {4 - D} \right)/2}} - a_{pc}^{{ * {{\left( {4 - D} \right)} /2}}} } \right) + \frac{2}{2 - D}\left( {A_{l}^{{ * \left( {2 - D} \right)/2}} - a_{pc}^{{ * {{\left( {2 - D} \right)} / 2}}} } \right)a_{pc}^{ * 2} } \right] \hfill \\ & -\frac{2}{{\left( {a_{ec}^{ * } - a_{pc}^{ * } } \right)^{3} }}\left[ \begin{aligned} \frac{2}{8 - D}\left( {A_{l}^{{ * \left( {8 - D} \right)/2}} - a_{pc}^{{ * {{\left( {8 - D} \right)} / 2}}} } \right) - \frac{6}{6 - D}\left( {A_{l}^{{ * \left( {6 - D} \right)/2}} - a_{pc}^{{ * {{\left( {6 - D} \right)} /2}}} } \right)a_{pc}^{ * } + \hfill \\ \frac{6}{4 - D}\left( {A_{l}^{{ * \left( {4 - D} \right)/2}} - a_{pc}^{{{{*\left( {4 - D} \right)} / 2}}} } \right)a_{pc}^{ * 2} - \frac{2}{2 - D}\left( {A_{l}^{{ * \left( {2 - D} \right)/2}} - a_{pc}^{{ * {{\left( {2 - D} \right)} /2}}} } \right)a_{pc}^{ * 3} \hfill \\ \end{aligned} \right] \hfill \\ \end{aligned} \right\} \hfill \\ \end{aligned} $$

Quartz, notable for its high hardness and content within rock, plays a significant role in influencing both drill bit wear and the rock abrasiveness. The actual wear-involved contact area between the asperity and drill bit, given the quartz content in the rock, is calculated as follows:

15 $$ A^{\prime}_{r} = \omega A_{r} $$

### Sliding friction of a PDC bit breaking rock

**Figure 4:**
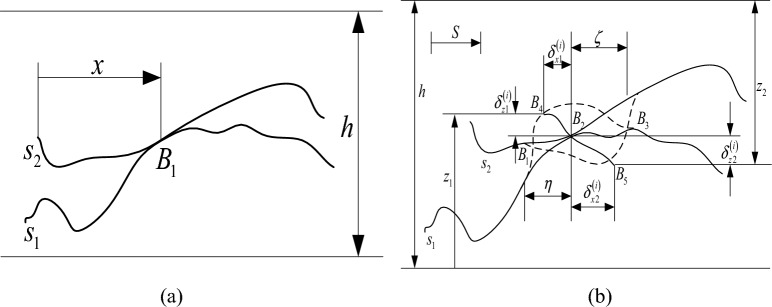
Sliding contact of microprotrusions.

As shown in Fig. 4 (a), at the minimum scale considered, a certain asperity pair $i$, which is in contact with point $B_{1}$ at a certain time, moves to point $B_{3}$ on surface $S_{2}$ in contact with point $B_{1}$ after a certain time relative to its sliding distance $S$, reaching the state shown in Fig. 4 (b). The dotted line in the figure represents the contour shape when it is not deformed, and the contact surface is represented by $A_{i}$; when elastic‒plastic deformation is not considered, the points where $B_{4}$ and $B_{5}$ are in contact originally become the points where $B_{2}$ is in contact due to elastic‒plastic deformation. If the deformation is $\delta_{1}^{\left( i \right)}$ and $\delta_{2}^{\left( i \right)}$, respectively, and the projections in the horizontal direction $\left( x \right)$ and the vertical direction $\left( z \right)$ are $\delta_{x1}^{\left( i \right)}$ and $\delta_{z1}^{\left( i \right)}$ and $\delta_{x2}^{\left( i \right)}$ and $\delta_{z2}^{\left( i \right)}$, respectively, the vertical distance between the two surfaces will change due to the deformation and the relative sliding along the contact surface. The change is derived from $t$:

16 $$ V_{z} = V_{x} \frac{{\partial z_{1} }}{{\partial x_{1} }} - \frac{{\partial \delta_{z}^{\left( i \right)} }}{\partial t} $$

The asperity has both mechanical deformation resistance (perpendicular to the microcontact surface) and molecular adhesion resistance (parallel to the microcontact surface) in the contact area. Therefore, the total contact pressure $p_{T}$ is not necessarily perpendicular to the contact surface and can be decomposed into the mechanical deformation resistance $q$ and molecular adhesion resistance $p$, namely:

17 $$ p_{T}^{\left( i \right)} \left( {x,y,z} \right) = p^{\left( i \right)} \left( {x,y,z} \right) + q^{\left( i \right)} \left( {x,y,z} \right) $$

In the formula, the projections of $p_{1}^{\left( i \right)}$ and $p_{2}^{\left( i \right)}$ and $q_{1}^{\left( i \right)}$ and $q_{2}^{\left( i \right)}$ in the corresponding directions are equal, and those in the opposite directions can be obtained as formula (18):

18 $$ V_{z} = V_{x} \frac{{p_{z1}^{\left( i \right)} }}{{p_{x1}^{\left( i \right)} }} - \frac{{\partial \delta_{z}^{\left( i \right)} }}{\partial t} $$

19 $$ V_{z} = V_{x} \frac{{q_{x1}^{\left( i \right)} }}{{p_{z1}^{\left( i \right)} }} - \frac{{\partial \delta_{z}^{\left( i \right)} }}{\partial t} $$

By integrating the sides of formula (18) and formula (19) on the spatial surface $A_{i}$ of the microcontact zone, we can obtain the constant $C$, where $0 < C < 1$ and $C$ simplifies to:

20 $$ R_{z1} = \frac{{CA_{r} }}{{A_{r} }}R_{x1} = CR_{x1} $$

21 $$ T_{x1} = \frac{{CA_{r} }}{{A_{r} }}T_{z1} = CT_{z1} $$

Since $F_{p} = R_{z1} + T_{z1}$, adhesion is caused mainly by plastic deformation, the adhesive shear strength is $\tau$, and the adhesive resistance can be expressed as the product of the adhesive shear strength and the plastic contact area $A_{rp}$. Therefore, the sliding friction force $F_{h}$ between the formation and the PDC bit surface is:

22 $$ F_{h} = CF_{p} + \left( {1 - C^{2} } \right)\tau A_{rp} $$

According to the fractal features of rough surfaces and classical contact mechanics theory, the plastic contact area is obtained and substituted into formula (22). Finally, the sliding friction force $F_{h}$ between the formation and the PDC bit surface is obtained:

23 $$ \left\{ \begin{aligned} & F_{h} = CF_{p} + \left( {1 + C^{2} } \right)\tau A_{a} A_{r}^{ * } \left( {\frac{{DG^{ * 2} }}{{\left( {2-D} \right)A_{r}^{ * } }}\left( {\frac{{k\sigma_{y} }}{{2E^{ * } }}} \right)^{{{2 /{1-D}}}} } \right)^{{{{2-D} / 2}}} \psi^{{{{\left( {D-2} \right)^{2} } /4}}} \hfill \\ & f_{h} = \frac{{F_{h} }}{{F_{p} }} \hfill \\ \end{aligned} \right. $$

where $F_{h}$ is the sliding friction force and $f_{h}$ is the sliding friction coefficient. $A_{r}^{ * } = {{A_{r} }/ {A_{a} }}$, $G^{ * } = {G}/ {A_{a}^{1/2}}$, and $A_{a}$ is the nominal contact area.

### Surface temperature rise function of the friction pair under sliding friction conditions

A high temperature increases the contact area and friction coefficient between the bit and formation surface, affecting friction. This section examines the temperature rise and overall distribution at the contact surface of a PDC bit and formation during sliding friction. Carslaw and Jaeger reported that for a circular microcontact with a radius of $r$, the friction temperature rise conforms to the following function^16^:

24 $$ T_{{\text{s}}} \left( {r^{\prime\prime}} \right) = \frac{qr}{\varepsilon }\int_{0}^{\infty } {J_{0} \left( {\lambda r^{\prime\prime}} \right)J_{1} \left( {\lambda r} \right)\frac{d\lambda }{\lambda }} $$

where $r^{\prime\prime}$ is the radial distance from the center of the microcontact point; $\varepsilon$ is the thermal conductivity; $q$ is the heat transferred to the surface; and $J_{0}$ and $J_{1}$ are the first zero-order and first-order Bessel functions, respectively.

Komvopoulos used the Gaussian distribution function as the approximate expression of formula (24). That is, the temperature distribution on the microcontact point is^17^:

25 $$ T_{{\text{s}}} \left( {r^{\prime\prime}} \right) = T_{{{\text{s}},{\text{M}}}} \exp \left[ { - \frac{{\left( {r^{\prime\prime}} \right)}}{{2\sigma_{T}^{2} }}} \right] $$

where $\sigma_{T}$ is the standard deviation of the Gaussian temperature distribution in the radial direction.

$\phi_{{\text{s}}} \left( {T_{{\text{s}}} } \right)$ is used to represent the temperature rise distribution density function of a single elastic microcontact area. Because $r = \left( {{a \mathord{\left/ {\vphantom {a \pi }} \right. \kern-0pt} \pi }} \right)^{{{1 \mathord{\left/ {\vphantom {1 2}} \right. \kern-0pt} 2}}}$, the temperature rise distribution density function of a single microcontact point is:

26 $$ \phi_{{\text{s}}} \left( {T_{{\text{s}}} } \right) = \left\{ \begin{aligned} &\frac{2\xi }{{T_{{\text{s}}} }}\;\;\;\;T_{{\text{s,m}}} \le T_{{\text{s}}} \le T_{{\text{s,M}}} \hfill \\ & 0\;\;\;\;\;\;T_{{\text{s}}} < T_{{\text{s,m}}} ,T_{{\text{s}}} > T_{{\text{s,M}}} \hfill \\ \end{aligned} \right. $$

According to the W-M contact model, the expressions of the temperature distribution density function $\phi \left( {T_{{\text{s}}} } \right)$ and the temperature rise cumulative distribution function $F\left( {T_{{\text{s}}} } \right)$ (that is, the real contact area where the temperature rise is greater than $T_{{\text{s}}}$) on the real contact surface are:

27 $$\phi \left( {{T_{\text{s}}}} \right) = \left\{ {\begin{array}{ll}   {\left( {\pi  - 2} \right)\xi \frac{1}{{{T_{{\text{s,max}}}}}}}& \quad {0 \leqslant {T_{\text{s}}} \leqslant \frac{2}{\pi }{T_{{\text{s,max}}}}} \\   {2\xi \left( {\frac{1}{{{T_{\text{s}}}}} - \frac{1}{{{T_{{\text{s,max}}}}}}} \right)}& \quad {\frac{2}{\pi }{T_{{\text{s,max}}}} < {T_{\text{s}}} < {T_{{\text{s,max}}}}} \\   0 &\quad {{T_{\text{s}}} < 0,{T_{\text{s}}} > {T_{{\text{s,max}}}}} \end{array}} \right.$$

28 $$\begin{aligned}  F\left( {{T_{\text{s}}}} \right) & = \int_{{T_{\text{s}}}}^\infty  {\phi \left( {{T_{\text{s}}}} \right)} {\text{d}}{T_{\text{s}}} \hfill \\   & = \left\{ {\begin{array}{ll}  1& \quad {{T_{\text{s}}} \leqslant 0} \\   {\left( {\pi  - 2} \right)\xi \left( {\frac{2}{\pi } - \frac{{{T_{\text{s}}}}}{{{T_{{\text{s,max}}}}}}} \right) + 2\xi \left[ {\ln \left( {\frac{\pi }{2}} \right) - \frac{{\pi  - 2}}{\pi }} \right]}& \quad {0 < {T_{\text{s}}} < \frac{2}{\pi }{T_{{\text{s,max}}}}} \\   {2\xi \left[ {\ln \left( {\frac{{{T_{{\text{s,max}}}}}}{{{T_{\text{s}}}}}} \right) - \frac{{{T_{{\text{s,max}}}} - {T_{\text{s}}}}}{{{T_{{\text{s,max}}}}}}} \right]}& \quad {\frac{2}{\pi }{T_{{\text{s,max}}}} < {T_{\text{s}}} < {T_{{\text{s,max}}}}} \\   0& \quad{{T_s} \geqslant {T_{s,\max }}} \end{array}} \right. \hfill \\ \end{aligned}$$

$T_{c}$ is the characteristic temperature rise, which reflects the maximum contact temperature rise caused by the friction between the drill bit and the formation under specific working conditions. The expression is:

29 $$ T_{c} = \frac{{f_{h} E^{ * } nRA_{a}^{{{1 \mathord{\left/ {\vphantom {1 2}} \right. \kern-0pt} 2}}} }}{{\sqrt \pi \left( {\varepsilon_{1} + \varepsilon_{2} } \right)}} $$

In the above formula, $T_{{\text{s,max}}}$ is the maximum temperature rise on the entire real contact surface; $T_{c}$ is the characteristic temperature rise; $f_{h}$ is the sliding friction coefficient; $n$ is the rotation speed of the drill; $R$ is the radius of the drill bit; and $\varepsilon_{1}$ and $\varepsilon_{2}$ are the thermal conductivities of the drill bit and formation rock, respectively.

At this time, the real contact area of the temperature rise in the range of $\left[ {T_{{\text{s,a}}} ,T_{{\text{s,max}}} } \right]$ can be obtained by formula (30):

30 $$ A^{\prime\prime}_{r} = \int_{{T_{{\text{s,a}}} }}^{{T_{{\text{s,max}}} }} {\phi \left( {T_{s} } \right)} {\text{d}}T_{{\text{s}}} = F\left( {T_{{\text{s,max}}} } \right) - F\left( {T_{{\text{s,a}}} } \right) $$

In the above formula, $T_{{\text{s,a}}}$ is the initial temperature, and the temperature in the laboratory is 20 °C.

Therefore, the sliding friction force between the drill bit and the formation under the condition of thermal–mechanical coupling is:

31 $$ F_{h} = CF_{p} + \left( {1 + C^{2} } \right)\tau A^{\prime\prime}_{r} \left( {\frac{{DG^{ * 2} A_{a} }}{{\left( {2 - D} \right)A^{\prime}_{r} }}\left( {\frac{{k\sigma_{y} }}{{2E^{ * } }}} \right)^{{\frac{2}{1 - D}}} } \right)^{{\frac{2 - D}{2}}} \psi^{{\frac{{\left( {D - 2} \right)^{2} }}{4}}} $$

## Evaluation index of formation abrasiveness based on the fractal dimension of the rock surface

### Rock surface morphology test

The roughness of rock surfaces can be measured by the fractal dimension, which impacts drill wear. This study uses SEM to analyze and quantify the surface morphology of rock cores to determine their fractal dimension. The surface morphology remains consistent across different magnifications, so only images at 3000 × magnification are shown^18^.

**Figure 5:**
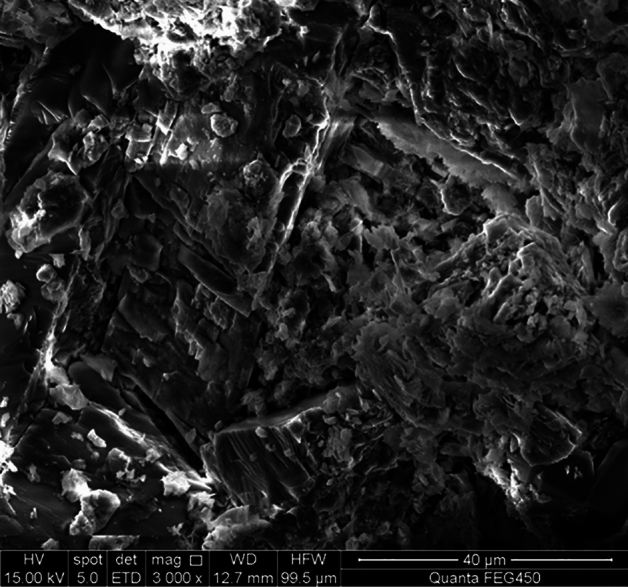
The core surface × 3000.

**Figure 6:**
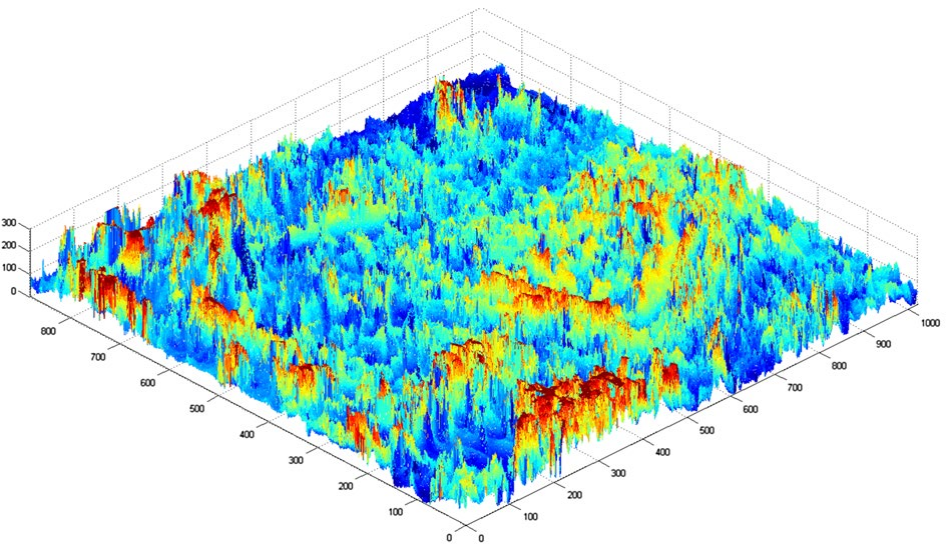
Three-dimensional reconstruction.

The grayscale values illustrated in the scanning electron microscope image presented in Fig. 5 are obtained through the methodology outlined in "Three-dimensional numerical reconstruction of the rock surface morphology" section. These values are then employed in MATLAB to generate a three-dimensional visualization, as shown in Fig. 6.

**Figure 7:**
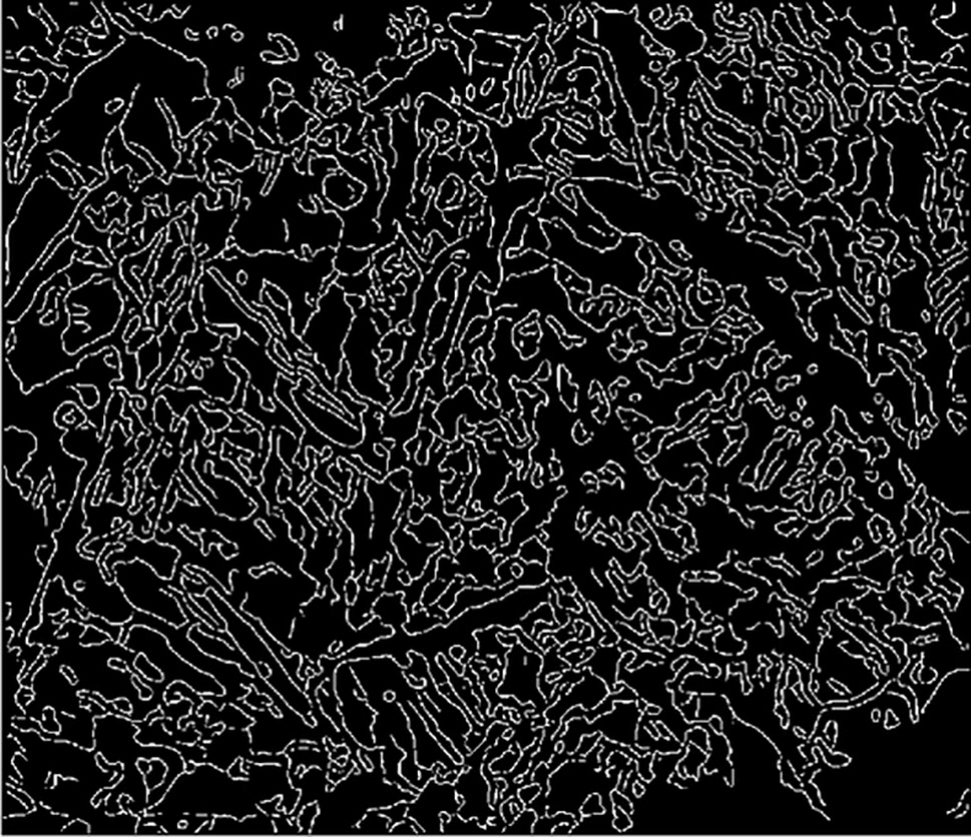
Core surface binary image.

The box-counting method is utilized for the computation of the fractal dimension by digitizing the three-dimensional rock surface depicted in Fig. 6. This transformation results in a two-dimensional binary image consisting of binary digits (0 and 1). The analysis of this binary image enables the calculation of the fractal dimension for the digitized representation, as illustrated in Fig. 7.

The box-counting dimension is assessed by calculating the number of pixels covered by squares of varying side lengths. The number of squares that are not empty is represented by $N\left( \varepsilon \right)$, where $\varepsilon$ indicates the scale of measurement or the pixel length of each small square. Within a double-logarithmic coordinate system $\ln \left[ {N\left( \varepsilon \right)} \right] - \ln \left( \varepsilon \right)$, the relationship between the scale of measurement and the count of nonempty squares is analyzed. By utilizing the least squares method, a linear fit is established, and the fractal dimension $D$ is determined by the absolute slope of the resulting line^19^.

### PDC bit wear formula

Archard proposed a formula for calculating adhesive wear. Considering that not all bonding points form hemispherical wear debris, the adhesive wear constants $k_{h}$ and $k_{h} \ll 1$ are introduced, and the Archard formula is^20^:

32 $$ \frac{{{\text{d}}V}}{{{\text{d}}s}} = k_{h} \frac{{F_{h} }}{{3\sigma_{y} }} $$

where $\frac{{{\text{d}}V}}{{{\text{d}}s}}$ is the wear volume generated by unit displacement and $\sigma_{y}$ is the compressive yield limit of the PDC material.

During the interaction between a formation–bit friction pair, the actual contact area emerges from the interplay of normal and tangential forces due to both normal and shear stresses. The yield stress correlates with the normal stress $\sigma_{n}$ from the normal load and the shear stress $\tau_{n}$ from the tangential load^21^. Wear from plastic and elastic contacts is assessed separately using the plastic contact wear coefficient $k_{p}$ and the elastic contact wear coefficient $k_{e}$, respectively. Additionally, the hard particle content and frictional heating of the rock are accounted for in the wear calculation. The wear can be expressed as:

33 $$ V = \left[ {1 + \lambda_{n} f^{2} } \right]^{\frac{1}{2}} \left[ {k_{e} A^{\prime\prime}_{r} + \left( {k_{p} - k_{e} } \right)A_{rp} } \right]s $$

Within the formula, $A_{rp}$ represents the area of plastic contact, which is determined using formula (34).

34 $${A_{rp}} = {\left( {\frac{D}{{2 - D}}} \right)^{\frac{{2 - D}}{2}}}{\psi ^{\frac{{{{\left( {2 - D} \right)}^2}}}{4}}}A_r^{\frac{D}{{\prime \prime 2}}}a_c^{\frac{{2 - D}}{2}}$$

**Figure 8:**
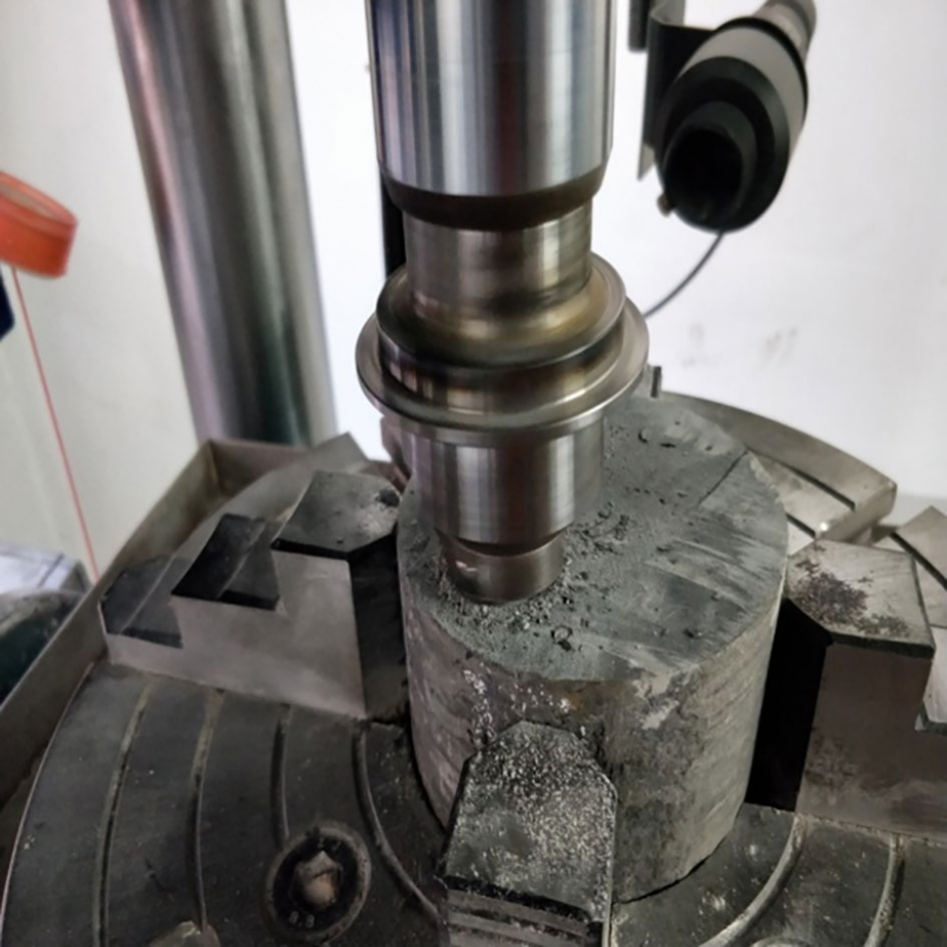
PDC bit friction and wear test.

The wear was tested using a self-developed rock abrasiveness test system , as shown in Fig. 8. The experimental parameters included an axial load of 0.5 kN, a rotation speed of 55 r/min, and 80 min of cumulative time for each group. The geometric parameters of the microdrill bit included a radius of 15.875 mm and a composite sheet radius of 6.5 mm, with a back angle of 20°. The measured material parameters of the diamond layer of the composite sheet were a density of 3.5 g/cm^3^, elastic modulus of 862 GPa, Poisson's ratio of 0.075, compressive strength of 1290 MPa, and shear strength of 1100 MPa.

By comparing the wear volume of the drill bit and the calculation results of the wear formula, the wear coefficients of the PDC drill bit are $k_{p} = 6.48 \times 10^{ - 3}$ and $k_{e} = 6 \times 10^{ - 4}$, respectively. Substituting this value into the wear formula (34), a comparison between the experimental and theoretical values is shown in Table 1.

**Table 1:** Results of the PDC bit tests.

Test number	Lithologic characteristics	Final temperature (℃)	Torque (N m)	Experimental wear volume (mm^3^)	Theoretical wear volume (mm^3^)	Coefficient of variation (%)
1	Rhyolite	79	12.06	0.831	0.609	15.70
2	Silty mudstone	76	8.79	0.204	0.165	2.76
3	Acidic volcanic eruption rock	74	9.81	0.815	0.919	7.35
4	Andesite	78	11	0.932	1.252	22.63
5	Sandy conglomerates	75	9.66	0.403	0.446	3.04
6	Mudstone	73	8.69	0.098	0.044	3.82
7	Fine sandy siltstone	76	10.11	0.416	0.398	1.27

The discrepancy between the predicted outcomes and theoretical wear calculations for the microdrill, as shown in Table 1, is typically less than 10%, underscoring the accuracy and utility of the formula. Although some results exhibit higher relative errors, there is a notable correlation between the experimental and theoretical findings regarding the impact of different rock types on drill bit wear, also highlighting the magnitude of rock abrasiveness.

### Formation–PDC bit abrasiveness evaluation index

Currently, drillability is commonly employed as a criterion for assessing drill bit selection^22^. It is generally believed that a drill bit exhibiting a higher rock-breaking efficiency is more adaptable than a drill bit exhibiting a lower rock-breaking efficiency, with little consideration given to the impact of drill bit wear on drilling efficiency. Hence, it is crucial to establish a comprehensive evaluation index for rock abrasiveness that accounts for both the rock-breaking efficiency of the drill bit and the wear parameters of the drill bit. This study utilizes the wear weight loss of the cutting tool of the rock cutting tool per unit volume as a metric for the rock abrasiveness index.

35 $$ \lambda_{abrasive} = \frac{\Delta W}{{\Delta V}} = \frac{{\text{weight loss of drill bit wear}}}{{\text{volume of fragmentized rock}}} $$

In the above formula, $\lambda_{abrasive}$ is the grindability index of rock, ${{{\text{mg}}} \mathord{\left/ {\vphantom {{{\text{mg}}} {{\text{cm}}^{{3}} }}} \right. \kern-0pt} {{\text{cm}}^{{3}} }}$.

This ratio-based index comprehensively evaluates both the rock-breaking efficiency and wear characteristics of the drill bit. It quantifies the wear resistance of the rock relative to that of the drill bit during the rock breaking process, providing an effective measure of both rock breaking performance and drill bit wear during drilling operations. The proposed method demonstrates good comparability and repeatability, making it a convenient tool for assessing drill bit consumption during drilling activities.

Under the same drilling conditions, the breaking volume of rock during drilling time $t$ can be calculated by the drillability grade $k_{dp}$ of the PDC bit:

36 $$ \Delta V = \frac{2.9t}{{2^{{k_{dp} }} }} $$

The experimental parameters of the PDC bit are substituted into the evaluation index of rock abrasiveness during sliding between the PDC bit and rock:

37 $$ \lambda_{abrasive} = 0.33\omega \cdot 2^{{k_{dp} }} \left\{ {\frac{{\left( {1 - \nu + 1.15 \times 10^{ - 6} E} \right) \cdot 2^{{\left( {3 - D} \right)}} }}{{961\left( {1 - \nu^{2} } \right)\cos \left( {\frac{17\pi + 36\varphi }{{72}}} \right)}} \cdot \left[ \begin{aligned} & \frac{{c\sqrt {1 + \cot^{2} \left( {\frac{19\pi - 36\varphi }{{72}}} \right)} }}{{8.7\sin \left( {\frac{19\pi - 36\varphi }{{72}}} \right)}} \hfill \\ & + h\left( {10^{ - 3} \rho g - 0.01} \right) \cdot \sin \left( {\frac{19\pi + 36\varphi }{{72}}} \right) \hfill \\ & \cdot \left[ {\cot \left( {\frac{19\pi - 36\varphi }{{72}}} \right) + 0.36} \right] \hfill \\ & \cdot \left[ {0.4\cot \left( {\frac{19\pi - 36\varphi }{{72}}} \right) - 0.125} \right] \hfill \\ \end{aligned} \right]} \right\}^{{\frac{2}{3 - D}}} $$

In this formula, $\rho$ is the drilling fluid density, ${{\text{g}} \mathord{\left/ {\vphantom {{\text{g}} {{\text{cm}}^{{3}} }}} \right. \kern-0pt} {{\text{cm}}^{{3}} }}$; $h$ is the depth of the well, ${\text{m}}$; $t$ is the drilling time, ${\text{s}}$; $E$ is the elastic modulus of the rock, ${\text{MPa}}$; $\nu$ is the Poisson's ratio of the rock; $c$ is the rock cohesion, ${\text{MPa}}$; $\varphi$ is the internal friction angle of the rock, ${\text{rad}}$; $\omega$ is the quartz content of the rock, ${\text{\% }}$; and $D$ is the fractal dimension of the rock surface roughness.

The abrasiveness of different types of rocks is calculated according to formula (37). Combined with the traditional experience of measuring abrasiveness by the internal friction angle at drilling sites^23^, a previously proposed classification scheme of rock abrasiveness is used to divide rocks into seven grades according to abrasiveness. The classification methods used are shown in Table 2. The parameters in formula (37) have a clear physical meaning, which can better reflect the essence of rock wear on PDC bits and improve the prediction accuracy of abrasiveness.

**Table 2:** Rock abrasiveness grading standard.

Level	Relative transmitting response wear rate (mg/cm^3^)	Angle of internal friction (°)	Abrasiveness classification
1	< 0.2	< 20	Low abrasiveness
2	0.2–0.89	20–38.9	Medium–low abrasiveness
3	0.89–1.65	38.9–40.4	Medium abrasiveness
4	1.65–2.5	40.4–50.2	Medium to high abrasiveness
5	2.5–4.5	50.2–51	Quite high abrasiveness
6	4.5–6.5	51–54	High abrasiveness
7	> 6.5	> 54	Very high abrasiveness

## Conclusion

1. This study examines the wear mechanisms during the interaction occurring between a PDC drill bit and a formation and establishes an abrasion evaluation method based on the fractal dimension of the rock surface topography. This method involves analyzing the three-dimensional digital model of scanning electron microscope images after rock drilling and utilizing the improved W-M function to assess the roughness of the rock surface, thereby determining its fractal dimension.
2. The microscopic contact area between the PDC bit and the formation is significantly smaller than the macroscopic contact area. By considering the distribution of microscopic convex points within a specific region of the two-body friction pair and the heat equation produced during sliding friction, the sliding friction force between the drill bit and the formation can be determined under thermo-mechanical coupling conditions.
3. A methodology for quantifying rock abrasiveness was developed based on the wear mass loss per unit volume of a rock cutting tool under sliding conditions, serving as an indicator of abrasiveness. The model incorporates parameters with clear physical significance and categorizes abrasivity into seven distinct grades based on various core experiment outcomes.

## Data Availability

The datasets used and/or analyzed in the current study are available from the corresponding author upon reasonable request.

## List of symbols

$$D$$: Fractal dimension
$$G$$: Scale coefficient
$E^{ * }$: The comprehensive elastic modulus of two contact surfaces (MPa)
$E$: Elastic modulus of rock (MPa)
$l$: Microband length (mm)
$a_{c}$: The critical area where plastic deformation occurs at the microcontact point (μm^2^)
$a_{l}$: Maximum asperity contact area of the formation–bit contact surface (μm^2^)
$a_{ec}$: Critical elastic deformation microcontact area (μm^2^)
$a_{pc}$: Critical plastic deformation microcontact area (μm^2^)
$F$: Total contact load of the asperity (MPa)
$F_{ep}$: Elastic load of asperity (MPa)
$F_{pp}$: Plastic load of the asperity (MPa)
$K_{f}$: Friction correction factor
$A_{r}$: Real contact area of the friction pair (μm^2^)
$A_{rp}$: Plastic contact area (μm^2^)
$A_{a}$: Nominal contact area (μm^2^)
$F_{h}$: Sliding friction force between the PDC bit and formation (MPa)
$\tau$: Adhesive shear strength (MPa)
$f_{h}$: Coefficient of sliding friction
$q$: The heat transferred to the surface (J)
$T_{{\text{s,max}}}$: Maximum temperature rise on the whole real contact surface (K)
$T_{c}$: Maximum contact temperature rise caused by the friction between the PDC bit and formation (K)
$n$: Rotation speed of drill (r/min)
$R$: Diameter of cone bit (mm)
$$t$$: Drilling time (h)
$$k_{dp}$$: Drillability
$c$: Rock cohesion (MPa)
$$k$$: Wear coefficient
$$h$$: Well depth (m)

## Greek symbols

$\sigma$: Mean square deviation of the contour curve
$\sigma_{y}$: Yield strength of the cutting teeth of the drill bit (MPa)
$\omega$: Percentage of quartz content (%)
$$\varepsilon$$: Thermal conductivity
$\sigma_{n}$: Normal stress (MPa)
$\tau_{n}$: Shear stress (MPa)
$\lambda_{abrasive}$: Shear stress (mg/cm^3^)
$$\nu$$: Poisson's ratio
$\varphi$: Internal friction angle (°)
$\rho$: Drilling mud density (g/cm^3^)

## References

[1] Chen P, Dai X, Shao F, Ozbayoglu E, Liu W, Wang J (2023). Review of PDC cutter—Rock interaction: Methods and physics. Geoenergy Sci. Eng..

[2] He D, Jia C, Zhao W, Xu F, Luo X, Liu W, Tang Y, Gao S, Zheng X, Li D, Zheng N (2023). Research progress and key issues of ultra-deep oil and gas exploration in China. Pet. Explor. Dev..

[3] Wang C, Li S, Zhang L (2019). Evaluation of rock abrasiveness class based on the wear mechanisms of PDC cutters. J. Petrol. Sci. Eng..

[4] Zhang S, Zhou Z, Gao Z, Cai X, Song W (2023). Analyzing the influence of Cerchar abrasiveness index on particle size distribution in ball milling based on multifractal theory. Powder Technol..

[5] Moradi M, Khosravi MH, Hamidi JK (2024). Introducing a new rock abrasivity index using a scaled down disc cutter. Rock Mech. Bull..

[6] Wang L, Guo K, Wu W (2023). Abrasivity measurement of brittle rock after thermal treatment. Measurement.

[7] Li Q, Li J, Duan L, Tan S (2021). Prediction of rock abrasivity and hardness from mineral composition. Int. J. Rock Mech. Min. Sci..

[8] Zhang S-R, She L, Wang C, Wang Y-J, Cao R-L, Li Y-L, Cao K-L (2021). Investigation on the relationship among the Cerchar abrasivity index, drilling parameters and physical and mechanical properties of the rock. Tunn. Undergr. Space Technol..

[9] Krishna Sastry KV, Seshagirirao V (2014). Optimization of drilling process parameters for minimizing surface roughness in carbon-carbon composite materials. Adv. Mater. Res..

[10] Boryczko A (2010). Distribution of roughness and waviness components of turned surface profiles. Metrol. Meas. Syst..

[11] Zhang Y, Li YE, Ku T (2021). Soil/rock interface profiling using a new passive seismic survey: Autocorrelation seismic interferometry. Tunn. Undergr. Space Technol. Incorporating Trenchless Technol. Res..

[12] Wu J-J (2000). Characterization of fractal surfaces. Wear.

[13] Mandelbrot BB, Passoja DANNE, Paullay AJ (1984). Fractal character of fracture surfaces of metals. Nature.

[14] Majumdar A, Bhushan B (1991). Fractal model of elastic-plastic contact between rough surfaces. J. Tribol..

[15] Wang S, Komvopoulos K (1994). A fractal theory of the interfacial temperature distribution in the slow sliding regime: Part I—elastic contact and heat transfer analysis. J. Tribol..

[16] De Jaeger P, T’Joen C, Huisseune H, Ameel B, De Schampheleire S, De Paepe M (2012). Assessing the influence of four cutting methods on the thermal contact resistance of open-cell aluminum foam. Int. J. Heat Mass Transf..

[17] Wang S, Komvopoulos K (1994). A fractal theory of the interfacial temperature distribution in the slow sliding regime: Part II—multiple domains, elastoplastic contacts and applications. J. Tribol..

[18] Wu K, He M, Yuan Z, Liu X, Luo B, Ma X, Ma C (2024). Characterizing rock transverse anisotropic spatial variations using digital drilling. Geoenergy Sci. Eng..

[19] Wang C, Wang X, Li S, Jiao Y, Yang R, Li G (2022). Evaluation method for tooth wear of roller bits based on the fractal dimension of the rock surface. J. Pet. Sci. Eng..

[20] Archard JF (1953). Contact and rubbing of flat surfaces. J. Appl. Phys..

[21] He J, Li S, Li X, Wang X, Guo J (2016). Study on the correlations between abrasiveness and mechanical properties of rocks combining with the microstructure characteristic. Rock Mech. Rock Eng..

[22] Wang H, He M, Zhao J, Zhang Y, Yang B (2023). Cutting energy characteristics for brittleness evaluation of rock using digital drilling method. Eng. Geol..

[23] Oparin VN, Tanaino AS (2015). A new method to test rock abrasiveness based on physico-mechanical and structural properties of rocks. J. Rock Mech. Geotech. Eng..

